# Effects of Premating Calcium and Phosphorus Supplementation on Reproduction Efficiency of Grazing Yak Heifers

**DOI:** 10.3390/ani11020554

**Published:** 2021-02-20

**Authors:** Jia Zhou, Jianxun Zhang, Benchu Xue, Shuangming Yue, Chao Yang, Bai Xue

**Affiliations:** 1 Animal Nutrition Institute, Sichuan Agricultural University, Chengdu 611130, China; zhoujia1@stu.sicau.edu.cn (J.Z.); zhangjianxun201206@163.com (J.Z.); xuebenchu@stu.sicau.edu.cn (B.X.); 2 Department of Bioengineering, Sichuan Water Conservancy Vocation College, Chengdu 611845, China; yueshuangming@stu.sicau.edu.cn (S.Y.); yangchaodongyi@163.com (C.Y.)

**Keywords:** bone metabolism, calcium, phosphorus, reproduction, yaks

## Abstract

**Simple Summary:**

This study was realized to explore the effects of calcium chloride (CaCl) and monocalcium phosphate (MCP) supplementation on the reproductive efficiency of grazing yak heifers. The body weight, serum markers of bone metabolism, and conception and calving rate of grazing yaks in control group and supplementary feeding groups were compared. The results revealed that supplementation with MCP but not CaCl could significantly improve the reproductive performance, possibly due to the improvement in body weight and bone phosphorus storage providing better estrous physiological conditions for grazing yak heifers. The findings of this study may be helpful and instructional to improve the reproductive efficiency of yaks on the Qinghai Tibet Plateau.

**Abstract:**

Reproductive efficiency is the main factor limiting yak production on the Tibet Plateau. The purpose of this study was to investigate the effect of supplementation with calcium chloride (CaCl) and monocalcium phosphate (MCP) for 30 days before breeding on body weight (BW) change, serum bone metabolism biomarkers, conception rate, and calving rate of grazing yaks. Ninety 3 year old yak heifers (153.05 ± 6.56 kg BW) were assigned to three treatments (*n* = 30 per treatment): grazing without supplementation (CONT), grazing plus calcium chloride supplementation (CaCl), and grazing plus monocalcium phosphate supplementation (MCP). Compared with the CONT group, supplementation with CaCl increased the serum concentrations of osteocalcin and decreased the alkaline phosphatase (ALP) levels (*p* < 0.05); supplementation with MCP increased the average daily gain (ADG), serum concentrations of phosphorus (P) and osteocalcin, conception rate, and calving rate (*p* < 0.05), whereas it decreased the serum concentrations of hydroxyproline, ALP, and calcitonin (*p* < 0.05). Both CaCl and MCP supplementation had no effect on serum calcium (Ca) concentration. The ADG, conception rate, and calving rate were higher in the MCP group than in the CaCl group (*p* < 0.05), while the serum concentrations of hydroxyproline and calcitonin were lower (*p* < 0.05). It could be concluded that premating supplementation with MCP increased the body weight gain and subsequent conception and calving rate of grazing yaks. Supplementation with MCP had a positive effect on body condition and bone metabolism, thus providing a better estrous condition for grazing yak heifers, which could contribute to enhancing reproduction efficiency.

## 1. Introduction

Yaks (*Bos grannies*) have successfully adapted to the harsh high-altitude (2500 to 6000 m) environment with long-term cold temperature (below zero degrees Celsius), playing an indispensable role in the alpine meadow grazing ecosystem as the key breed of the pasture–livestock industry on the Tibetan Plateau [1]. Currently, more than 90% of yaks in the world live in China, which provide local residents with meat, dairy products, service force, and fuel [2]. Unfortunately, compared to the vast majority of cattle living in the plains, yaks generally exhibit lower reproductive efficiency [3]. Unlike domestic cattle, grazing yaks are considered to be seasonal breeders with mating occurring only from July to October (warm season) [4]. The sexual maturity of yaks is relatively late, with male yaks first used at the age of 3 years and female yaks first calving after 4 years [5]. On the other hand, yaks also face the problem of low fertility. According to statistics, the average annual reproduction rate of adult yaks is less than 49%, and more than half of them calve once every 2 years or twice in 3 years [6]. In addition, female yaks can experience a long postpartum anestrus period, whereby approximately 90% of postpartum female yaks cannot be rutted during the breeding season in the same year [7,8]. Therefore, it is of great practical significance for the development of yak production to explore methods for improving reproductive performance.

Nutritional status and dietary nutrition levels are the crucial factors determining the reproduction of female yaks [8]. Yaks kept in zoos or farmhouses did not show obvious seasonal estrus characteristics [9], which may be related to the continuous supply of food. However, in the wild, grazing yaks have to go through the withering process of grass in the cold season (November to the following May), during which the shortage of available pasture cannot satisfy the nutritional requirement of yaks [10]. The inadequate intake of nutrients would lead to a depletion of body reserves and decline in body condition of pregnant yaks. This results in a long postpartum anestrus of those yaks, because they need much more time to recover from the poor body conditions after calving [9]. Nutritional supplementation has a positive effect on the body weight gain and body condition of grazing yaks, which could be helpful for improving reproductive performance. Previous studies reported that supplementation with concentrates during the cold season [11,12,13] and warm season [14,15] could enhance the growth potential of grazing yaks. The body condition and nutritional status of beef cows are related to age at first calving, duration of the postpartum interval after successive calving, and conception and pregnancy rate [16]. Regretfully, we only found one report showing a greater calving rate and a shorter postpartum anestrous interval in yak cows after supplementation with oat hay or barley straw during the cold season [17]. Moreover, data describing the effects of mineral supplementation on yak reproduction are even more limited.

The deficiency of calcium (Ca) and phosphorus (P) negatively influences many different steps of the reproduction process in mammals, from gamete maturation to fetal development [18,19]. As mentioned previously, if the intake of Ca and P is insufficient because of the shortage of forages in the cold season, the body reserves of Ca and P become depleted and the reproductive potential of yak cows is inhibited. In addition, studies in ruminants have implied that supplementation with Ca increased the proportion of cows inseminated [20] and supplementation with P improved the reproductive efficiency of grazing cattle [21]. We hypothesized that the deficiency of Ca and P is one of the factors limiting the reproductive efficiency of grazing yaks, and that supplementation with Ca and P affects the body reserves of Ca and P and, thus, the reproductive performance of grazing yak heifers. The objectives of this study were to explore if supplementation with calcium chloride (CaCl) and monocalcium phosphate (MCP) for 30 days before breeding is necessary for grazing yaks, as well as its relationship with Ca and P metabolism.

## 2. Materials and Methods

### 2.1. Animals and Treatments

The procedures for animal processing referred to Chinese Animal Welfare Guidelines, and the experimental protocol was approved by the Animal Care and Ethical Committee of Sichuan Agricultural University (#SCAUAC201408-3). The experiment was conducted at the summer pastures (altitude of 3100 m) of Animal Husbandry and Veterinary Institute of Haibei Prefecture (Qinghai Province, China) from June to July. The pasture composition included *Kobresia humilis*, *Stipa aliena*, *Leontopodium nanum*, *Potentilla multifida*, *Taraxacum brevirostre*, *Oxytropis deflexa*, and *Gentiana squarrosa*. The content of Ca and P in pasture is provided in Table 1. Ninety 3 year old Qinghai plateau yak heifers (153.05 ± 6.56 kg body weight (BW)) were selected and randomly assigned to three treatments with 30 yaks in each treatment as follows: the control treatment, i.e., grazing without supplementation (CONT), grazing with CaCl supplementation (14 g/day each yak) (CaCl), and grazing with MCP supplementation (30 g/day each yak) (MCP). The dosage of MCP was based on a previous study [22], and the daily intake of Ca per yak in the CaCl treatment was equal to that in the MCP treatment. After a 2 week transition period for yak heifers to adapt to supplementary feeding, the 30 day nutritional intervention was initiated. All yaks grazed in the same pasture and had free access to water between 7:00 a.m. and 6:00 p.m. CaCl and MCP were mixed into ground maize with 100 g/head/day and fed to yaks at 7:00 p.m., whereas yaks in CONT group received the same amount of ground maize. The actual daily intake of calcium (Ca) and phosphorus (P) among yaks in the experimental groups is shown in Table 1. All yak heifers were mixed with yak bulls for natural mating after the nutritional intervention.

### 2.2. Sample Collection

Approximately 10 mL of blood was withdrawn from the jugular vein from each yak into evacuated tubes without anticoagulant on day 30 before grazing in the morning. The tubes were centrifuged at 2500 rpm for 15 min at 4 °C to obtain serum samples. All serum samples were aliquoted and immediately frozen at −80 °C until analyzed.

All yak heifers were weighed with a platform scale and recorded on two consecutive days before morning grazing pre and post trial. Data related to conception, miscarriage, and calving were observed and recorded for each female yak until the next estrus.

### 2.3. Serum Analyses

The serum concentrations of Ca and P were measured with commercial kits using a Shimadzu CL-7200 Automatic Biochemical Analyzer (Shimadzu, Kyoto, Japan). The serum concentrations of hydroxyproline and activity of alkaline phosphatase (ALP) were also determined using commercially kits (Nanjing Jiancheng, Nanjing, China). In addition, osteocalcin and calcitonin in serum were measured using ELISA kits (Meimian, Yancheng, China). The manufacturer’s instructions were strictly followed to obtain accurate results.

### 2.4. Statistical Analysis

The experimental data related to growth performance and serum parameters between groups were analyzed using one-way ANOVA followed by a multiple range test; results are presented as means ± standard deviation (SD). The data related to conception rate and calving rate were evaluated using χ^2^ analysis. All statistical tests were performed using SPSS 19.0 software (SPSS Inc. Chicago, IL, USA). A *p*-value ≤ 0.05 was considered statistically significant.

## 3. Results

### 3.1. Body Weight

Data on the body weight of yak heifers are presented in Table 2. The average daily gain (ADG) in the MCP group was 48.94% (*p* < 0.05) and 32.70% (*p* < 0.05) higher than that in the CONT and CaCl groups, respectively. Supplementation with CaCl did not affect the body weight compared to the CONT group.

### 3.2. Serum Parameters

Supplementation with Ca and P altered serum parameters, as illustrated in Table 3. Compared with the CONT group, the serum osteocalcin concentrations were higher (*p* < 0.05) in the CaCl group, whereas the serum concentrations of P and osteocalcin were higher (*p* < 0.05), while hydroxyproline and calcitonin were lower (*p* < 0.05) in the MCP group; furthermore, the activity of ALP was lower in the CaCl and MCP groups (*p* < 0.05). The serum hydroxyproline concentrations were higher in the CaCl group than in the MCP group (*p* < 0.05).

### 3.3. Reproduction Performance

The conception and calving rates were higher in the MCP group than in the CONT and CaCl groups (*p* < 0.05), as shown in Table 4.

## 4. Discussions

In grazing livestock, phosphorus deficiency is one of the most common mineral deficiencies in the world [24]. As reported in previous studies, the feeding intake (day matter) of 3 year old yaks is 3.262 kg/day/head in the grassy period [25] which is in accordance with the predicted value of dry matter intake (DMI) of beef cows [26]. Thus, we could calculate the daily intake of P of grazing yak as 8.81 g according to the P content of forage in this period (Table 1). This result is between the recommendations of the National Research Council (NRC) [27] for 150 kg growing dairy cows with a daily gain of 300 g (8 g/head/day) to 400 g (10 g/head/day). Phosphorus and Ca metabolism is inextricably associated due to these two minerals playing a common role in bone formation. Here, we compared the recommended and daily Ca intake of grazing yaks using the same method and found that the daily Ca intake was significantly higher than the recommendation of the NRC [27] for 150 kg growing dairy cows with a daily gain of 400 g (27.9 vs. 19.0 g/head/day). In addition, the serum concentrations of P in the CONT (1.35 mmol/L) and CaCl group (1.49 mmol/L) were below the recommended level of 1.5 mmol/L [28]. The serum Ca concentrations of all yaks were in the recommended range (2.25–2.75 mmol/L) [26], which is consistent with the result of Fan et al. (2019) [29]. These results indicated that the problem of P deficiency in grazing yaks may not only occur in the cold season [29], but also at the beginning of the warm season. Supplementation with MCP had a mitigative effect, whereas CaCl did not.

P deficiency has negative effects on the growth efficiency, feed conversion rate, appetite, reproductive function, milk yield, and bone metabolism of beef cattle [30,31]. In this condition, a decrease in DMI would limit microbial activity, thus inhibiting the digestion of fiber and the synthesis of microbial protein, such that the supply of amino acids to the intestine and the growth performance of animals are reduced [28]. Therefore, supplementation with P through MCP could significantly increase the body weight change and average daily gain of grazing yak heifers. Supplementation with MCP provided both P and Ca, whereas supplementation of the same amount of Ca had no effect on the body weight change and reproductive efficiency of grazing yaks in the CaCl group. This is possible due to the fact that grasses can provide an adequate intake of Ca and maintain normal serum Ca levels for grazing yaks. Additionally, the intake adjustment of Ca/P ratio may also be involved, because the excess or deficiency of one mineral may affect the utilization of the other [32].

The allocation of nutrients to various body functions is often called nutrient distribution [33]. Due to the genetic effect of a long evolutionary process, herbivores are capable of transforming low-quality forages into meat, fur, or milk, as well as of storing nutrients during periods of excessive nutrition supply, which could be used for maintaining production at a later time [33]. Nevertheless, a long-term nutritional supply can still reduce productivity. Generally, the nutrients of cows are prioritized to maintain basal metabolism, feed intake, growth, and basic energy reserves, followed by pregnancy, lactation, and extra energy reserves, and finally estrous cycles, initiation of pregnancy, and excess energy reserves [34]. The energy reserves are manifested as body fat deposition and body weight gain. In reality, the weight loss of grazing yaks during the cold season (November to May) accounted for more than 25% of the weight gain in the same growing season [14]. The yaks in this experiment were in the compensatory growth stage [35], thereby recovering their body condition after undergoing feeding restrictions in the previous cold season. However, P deficiency limited the growth potential of yak heifers in this period. In this study, we found that the performance in terms of growth and reproduction in the MCP group was greater than that in the CONT and CaCl groups. It can be inferred that MCP supplementation could further develop the compensatory growth potential of grazing yaks, which may have a positive effect on the estrous initiation or follicular development of yak heifers. Body condition and nutrient intake control the onset of estrus by regulating pituitary activity in beef cattle [36]. Therefore, supplementation with MCP before mating could increase the conception rate and calving rate of grazing yak heifers by improving body condition and nutrient reserves.

It seems that serum Ca level is not recognized as a reliable marker of Ca status, as it is regulated by a variety of hormones including calcitonin and kept within a stable range [32,37]. The concentrations of serum calcitonin increase in response to hypercalcemia [38], thus decreasing serum Ca levels via inhibiting the resorption of bone Ca and increasing the efflux of urinary Ca [39]. In the current study, we found that serum concentrations of Ca and calcitonin were not affected by CaCl supplementation, which may be related to the mechanism of Ca homeostasis. On the other hand, the absorption and retention of Ca are generally stable or even decrease with increased concentrations of excess dietary Ca [40], which may be one of the reasons for CaCl supplementation having no effect on the performance of growth and reproduction for grazing yak heifers. Although calcium is a critical factor for oocyte maturation [41] and fertilization [18], we do not discuss these topics here.

Approximately 80% of P is stored in the bones and teeth, with the rest distributed in soft tissues. In adult mammals, P deficiency caused by insufficient P intake can mobilize bone P to maintain the normal requirements of physiological function. Contrarily, extra P intake can promote the P reserves in bone [26]. As a marker of bone turnover [42] and bone formation [43], osteocalcin is a bone-specific product secreted by mature osteoblasts, which is used to directly reflect osteoblast function [44]. Osteocalcin can bind strongly to hydroxyapatite to bridge the matrix and mineral fractions of bone tissue [45]. Serum levels of osteocalcin have been proven to be closely related to the bone formation rate [46], and they are positively correlated with serum P levels [47], which is consistent with the findings of this study, suggesting that bone formation could be improved in the MCP group. Previous studies showed that phosphorus-deficient diets could reduce bone formation to maintain serum levels of Ca and P in lactation cows [48] and decrease the serum osteocalcin levels in growing–finishing pigs [49]. Similarly, alkaline phosphatase (ALP) and hydroxyproline are markers of osteoblast activity [50] and bone resorption [51], respectively. Alkaline phosphatase is mainly derived from osteoblasts and participates in inhibiting bone mineralization, increasing bone mass, and enhancing bone metabolism [52]. Inadequate P intake can promote the secretion of parathyroid hormone to improve the proliferation of osteoblasts, which results in a great deal of ALP entering the blood, thereby increasing the serum activity of ALP [53]. In this study, a lower serum ALP activity was observed in the MCP group, which is in agreement with previous reports [54,55]. Hydroxyproline is a degradation product of bone collagen, metabolized in the liver before flowing through the blood and being excreted in urine [56,57]. Urinary hydroxyproline is usually regarded as a marker of bone resorption [58]. A decrease in serum hydroxyproline levels can be considered as weaker bone resorption metabolism, as seen in the MCP group. Bone is a metabolically active tissue, which relies on the balance between bone formation (osteoblasts) and bone resorption (osteoclasts) for continuous remodeling [58]. The results related to serum markers of bone metabolism in this study showed that supplementation with MCP tended to improve the amount of newly formed bone, which provided conditions for the reserves of P and other minerals stored in bone. The storage of minerals, especially P, may contribute to the subsequent estrous initiation and pregnancy of yak heifers.

In the current study, P deficiency in grazing yaks could be recognized, which may be a reason for the restriction of growth and reproduction. Despite P deficiency being found to alter estruses and lower conception rates of cows [59,60], excessive dietary levels of P were found to elevate serum P levels but not affect the reproductive performance [61,62]. We found that supplementation with MCP provided a better pregnancy precondition, including body condition and P reserves for grazing yak heifers, as well as achieved a better reproduction efficiency. The findings of this study suggest that supplementation with P is necessary for improving the growth performance and reproduction efficiency of grazing yak heifers.

## 5. Conclusions

The results of the current study reveal that supplementation with MCP can improve the body condition and P storage in bone of grazing yak heifers, which may create favorable physiological conditions for estrus and pregnancy. Our findings provide a new reproductive strategy for yak production on the Tibet Plateau.

## Figures and Tables

**Table 1:** The content ^1^ of calcium (Ca) and phosphorus (P) in pasture, calcium chloride (CaCl), and monocalcium phosphate (MCP; air-dried basis).

Items	CaCl	MCP	Pasture
Dry matter (%)	99.52	98.20	92.87
Ca (%)	35.98	16.37	0.92
P (%)	0.00	22.14	0.27
Supplementation intake (g/day)	14.00	30.00	-
Daily intake of Ca (g/day)	5.04	4.91	-
Daily intake of P (g/day)	0.00	6.64	-

^1^ The content of Ca and P was analyzed according to the Association of Official Analytical Chemists (AOAC) [23].

**Table 2:** Effects of calcium (Ca) and phosphorus (P) supplementation on the body weight of yak heifers (mean ± SD). CONT, control.

Items	CONT	CaCl	MCP
Initial weight (kg)	153.2 ± 5.7	155.4 ± 7.33	150.6 ± 6.7
Final weight (kg)	162.6 ± 7.1	166.0 ± 7.65	164.6 ± 8.8
Body weight change (kg)	9.4 ± 1.9 ^a^	10.6 ± 2.4 ^a^	14.1 ± 2.5 ^b^
Average daily gain (g/d)	313.3 ± 65.6 ^a^	351.7 ± 80.7 ^a^	466.7 ± 82.03 ^b^

Data with superscript letters within rows are significantly different (*p* < 0.05).

**Table 3:** Effects of calcium (Ca) and phosphorus (P) supplementation on serum parameters of yak heifers (mean ± SD).

Items ^1^	CONT	CaCl	MCP
Serum P (mmol/L)	1.35 ± 0.10 ^a^	1.49 ± 0.08 ^a,b^	1.79 ± 0.07 ^b^
Serum Ca (mmol/L)	2.32 ± 0.06	2.33 ± 0.08	2.32 ± 0.03
Hydroxyproline (μg/mL)	1.73 ± 0.04 ^b^	1.72 ± 0.04 ^b^	1.65 ± 0.05 ^a^
ALP (U/L)	52.21 ± 1.02 ^b^	49.86 ± 0.72 ^a^	49.74 ± 0.45 ^a^
Osteocalcin (ug/L)	1.80 ± 0.07 ^a^	1.94 ± 0.08 ^b^	1.96 ± 0.07 ^b^
Calcitonin (ng/L)	145.47 ± 1.16 ^b^	143.19 ± 2.03 ^b^	139.26 ± 1.38 ^a^

^1^ Serum P: serum phosphorus; Serum Ca: serum calcium; ALP: alkaline phosphatase. Data with superscript letters are significantly different (*p* < 0.05).

**Table 4:** Effects of calcium (Ca) and phosphorus (P) supplementation on reproductivity of yak heifers.

Items	CONT	CaCl	MCP
Number of yak heifers	30	30	30
Conceived yaks (No. ^1^)	19	19	25
Conception rate (%)	63.33 ^a^	63.33 ^a^	83.33 ^b^
Aborted yaks (No.)	1	2	1
Calving yaks (No.)	18	17	24
Calving rate (%)	60 ^a^	56.67 ^a^	80 ^b^

^1^ No: Number. Data with superscript letters are significantly different (*p* < 0.05).

## Data Availability

The data presented in this study are available on request from the corresponding author.

## References

[1] Ding X.Z., Guo X., Yan P., Liang C.N., Bao P.J., Chu M. (2012). Seasonal and nutrients intake regulation of lipoprotein lipase (LPL) activity in grazing yak (Bos grunniens) in the Alpine Regions around Qinghai Lake. Livest. Sci..

[2] Guo X., Long R., Kreuzer M., Ding L., Shang Z., Zhang Y., Yang Y., Cui G. (2014). Importance of functional ingredients in yak milk-derived food on health of Tibetan nomads living under high-altitude stress: A review. Crit. Rev. Food Sci..

[3] Prakash B.S., Sarkar M., Mondal M. (2008). An update on reproduction in yak and mithun. Reprod. Domest. Anim..

[4] Yu S.J., Huang Y.M., Chen B.X. (1993). Reproductive patterns of the yak. II. Progesterone and oestradiol-17 beta levels in plasma and milk just before the breeding season; also during normal and short oestrous cycles. Br. Vet. J..

[5] Mann G.E. (1993). Reproduction in the yak. Br. Vet. J..

[6] Lan D., Xiong X., Huang C., Mipam T.D., Li J. (2016). Toward Understanding the Genetic Basis of Yak Ovary Reproduction: A Characterization and Comparative Analyses of Estrus Ovary Transcriptiome in Yak and Cattle. PLoS ONE.

[7] Fu M., Xiong X.R., Lan D.L., Li J. (2014). Molecular characterization and tissue distribution of estrogen receptor genes in domestic yak. Asian Aust. J. Anim. Sci..

[8] Xiao X., Zi X.D., Niu H.R., Xiong X.R., Zhong J.C., Li J., Wang L., Wang Y. (2014). Effect of addition of FSH, LH and proteasomeinhibitor MG132 to in vitro maturation medium on the developmental competence of yak (Bos grun-niens) oocytes. Reprod. Biol. Endocrin..

[9] Zi X.D. (2003). Reproduction in female yaks (Bos grunniens) and opportunities for improvement. Theriogenology.

[10] Long R.J., Ding L.M., Shang Z.H., Gao X.H. (2008). The yak grazing system on the Qinghai-tibetan plateau and its status. Rangel. J..

[11] Zhou J., Yue S., Peng Q., Wang L., Wang Z., Xue B. (2020). Metabonomic Responses of Grazing Yak to Different Concentrate Supplementations in Cold Season. Animals.

[12] Long R.J., Dong S.K., Wei X.H., Pu X.P. (2005). The effect of supplementary feeds on the body weight of yaks in cold season. Livest. Prod. Sci..

[13] Ding L.M., Chen J.Q., Long R.J., Malcolm J.G. (2015). Blood hormonal and metabolite levels in grazing yak steers undergoing compensatory growth. Anim. Feed Sci. Tech..

[14] Xue B., Zhao X.Q., Zhang Y.S. (2005). Seasonal changes in weight and body composition of yak grazing on alpine-meadow grassland in the Qinghai-Tibetan plateau of China. J. Anim. Sci..

[15] Xie R., Zheng Q., Luo G. (2004). Finishing Effect of Maiwa Yak by Supply Feed in Warm Season. J. Grass Feed. Livest..

[16] Diskin M.G., Kenny D.A. (2016). Managing the reproductive performance of beef cows. Theriogenology.

[17] Long R.J., Zhang D.G., Wang X., Hu Z.Z., Dong S.K. (1999). Effect of strategic feed supplementation on productive and reproductive performance in yak cows. Prev. Vet. Med..

[18] De Clercq K., Vriens J. (2018). Establishing life is a calcium-dependent TRiP: Transient receptor potential channels in reproduction. Biochim. Biophys. Acta. Mol. Cell Res..

[19] Call J.W., Butcher J.E., Blake J.T., Smart R.A., Shupe J.L. (1978). Phosphorus influence on growth and reproduction of beef cattle. J. Anim. Sci..

[20] Pinedo P., Velez J., Solano G., Rodriguez N., Naves J., Schuenemann G.M., Risco C. (2017). Effect of oral calcium administration on the cure and reproductive performance of Holstein cows diagnosed with puerperal metritis. J. Dairy Sci..

[21] Dunn T.G., Moss G.E. (1992). Effects of nutrient deficiencies and excesses on reproductive efficiency of livestock. J. Anim. Sci..

[22] Jubb T.F., Crough K.F. (1988). Phosphorus supplementation of cattle. Aust. Vet. J..

[23] AOAC International (2002). Official Methods of Analysis.

[24] McDowell L.R. (2003). Minerals in Animal and Human Nutrition.

[25] Liu S.J. (1997). Determination of Feed Intake of Grazing Yaks in Different Phenological Periods. Yak Production in Central Asian Highlands: Proceedings of the Second International Congress on Yak, 1–6 September 1997, Xining, China.

[26] NRC (2016). Nutrient Requirements of Beef Cattle.

[27] NRC (2001). Nutrient Requirements of Dairy Cattle.

[28] Yoshihara Y., Mizuno H., Yasue H., Purevdorj N., Ito T.Y. (2013). Nomadic grazing improves the mineral balance of livestock through the intake of diverse plant species. Anim. Feed Sci. Tech..

[29] Fan Q., Wanapat M., Hou F. (2019). Mineral Nutritional Status of Yaks (Bos Grunniens) Grazing on the Qinghai-Tibetan Plateau. Animals.

[30] Shupe J.L., Butcher J.E., Call J.W. (1988). Clinical signs and bone changes associated with phosphorus deficiency in beef cattle. Am. J. Vet. Res..

[31] Underwood E.J., Suttle N.F. (2000). The mineral nutrition of livestock, 3rd ed. Br. J. Nutr..

[32] Crenshaw T.D. (2001). Calcium, Phosphorus, Vitamin D, and Vitamin K in Swine Nutrition. Swine Nutrition.

[33] Short R.E., Adams D.C. (1988). Nutritional and Hormonal Interrelationships in Beef Cattle Reproduction. Can. J. Anim..

[34] Short R.E., Bellows R.A., Staigmiller R.B., Berardinelli J.G., Custer E.E. (1990). Physiological mechanisms controlling anestrus and infertility in postpartum beef cattle. J. Anim. Sci..

[35] Ding L.M., Long R.J., Yang Y.H., Xu S.H. (2007). Behaviour responses by yaks, in different physiological states (lactating, dry or replacement heifer), when grazing natural pasture in spring (dry and germinating) season of Qinghai-Tibetan plateau. Appl. Anim. Behav. Sci..

[36] Rutter L.M., Randel R.D. (1984). Postpartum nutrient intake and body condition: Effect on pituitary function and onset of estrus in beef cattle. J. Anim. Sci..

[37] Heaney R.P. (1990). Estrogen-calcium interactions in the postmenopause: A quantitative description. Bone Miner..

[38] Findlay D.M., Sexton P.M. (2004). Calcitonin. Growth Factors.

[39] Murray R.D., Horsfield J.E., McCormick W.D., Williams H.J., Ward D. (2008). Historical and current perspectives on the treatment, control and pathogenesis of milk fever in dairy cattle. Vet. Rec..

[40] González-Vega J.C., Liu Y., Mccann J.C. (2016). Requirement for digestible calcium by eleven- to twenty-five-kilogram pigs as determined by growth performance, bone ash concentration, calcium and phosphorus balances, and expression of genes involved in transport of calcium in intestinal and kidney cells. J. Anim. Sci..

[41] Carroll J. (2001). The initiation and regulation of Ca^2+^ signalling at fertilization in mammals. Semin. Cell Dev. Biol..

[42] Ivaska K.K., Hentunen T.A., Vaaraniemi J., Ylipahkala H., Pettersson K., Vaananen H.K. (2004). Release of intact and fragmented osteocalcin molecules from bone matrix during bone resorption in vitro. J. Biol. Chem..

[43] Lian J., Stewart C., Puchacz E., Mackowiak S., Shalhoub V., Collart D., Zambetti G., Stein G. (1989). Structure of the rat osteocalcin gene and regulation of vitamin D-dependent expression. Proc. Natl. Acad. Sci. USA.

[44] Grimm M., Müller A., Hein G., Fünfstück R., Jahreis G. (2001). High phosphorus intake only slightly affects serum minerals, urinary pyridinium crosslinks and renal function in young women. Eur. J. Clin. Nutr..

[45] Ritter N.M., Farach-Carson M.C., Butler W.T. (1992). Evidence for the formation of a complex between osteopontin and osteocalcin. J. Bone Miner. Res..

[46] Delmas P.D., Malaval L., Arlot M. (1985). Serum bone-Gla-protein compared to bone histomorphometry in endocrine diseases. Bone.

[47] Zhang X.Y., He J.W., Fu W.Z., Liu Y.J., Zhang Z.L. (2016). Associations of Serum Osteocalcin and Polymorphisms of the Osteocalcin Gene with Bone Mineral Density in Postmenopausal and Elderly Chinese Women. J. Nutr. Nutr..

[48] Peterson A.B., Orth M.W., Goff J.P., Beede D.K. (2005). Periparturient responses of multiparous Holstein cows fed different dietary phosphorus concentrations prepartum. J. Dairy Sci..

[49] Sørensen K.U., Kruger M.C., Hansen-Møller J., Poulsen H.D. (2018). Bone biochemical markers for assessment of bone responses to differentiated phosphorus supply in growing-finishing pigs. J. Anim. Sci..

[50] Eleniste P.P., Huang S., Wayakanon K., Largura H.W., Bruzzaniti A. (2014). Osteoblast differentiation and migration are regulated by dynamin GTPase activity. Int. J. Biochem. Cell Biol..

[51] Devkota B., Itagaki K., Kim D., Sasaki K., Osawa T., Furuhama K., Yamagishi N. (2012). Relationship between sex hormone fluctuations and biomarkers of bone resorption in bovine plasma during the oestrous cycle. Vet. J..

[52] Hurwitz S., Fishman S., Talpaz H. (1987). Model of plasma calcium regulation: System oscillations induced by growth. Am. J. Physiol..

[53] Zhao L., Li M., Sun H. (2019). Effects of dietary calcium to available phosphorus ratios on bone metabolism and osteoclast activity of the OPG /RANK/RANKL signalling pathway in piglets. J. Anim. Physiol. Anim. Nutr..

[54] Boyd R.D., Hall D., Wu J.F. (1983). Plasma alkaline phosphatase as a criterion for determining biological availability of phosphorus for swine. J. Anim. Sci..

[55] Koch M.E., Mahan D.C. (1986). Biological characteristics for assessing low phosphorus intake in finishing swine. J. Anim. Sci..

[56] Lowry M., Hall D., Brosnan J. (1985). Hydroxyproline metabolism by the rat kidney: Distribution of renal enzymes of hydroxyproline catabolism and renal conversion of hydroxyproline to glycine and serine. Metabolism.

[57] Kivirikko K.I. (1970). Urinary excretion of hydroxyproline in health and disease. Int. Rev. Connect. Tissue Res..

[58] Seibel M.J. (2005). Biochemical markers of bone turnover: Part I: Biochemistry and variability. Clin. Biochem. Rev..

[59] Hignett S.L., Hignett P.G. (1952). Hignett. The influence of nutrition on reproductive efficiency in cattle. II. The effect of the phosphorus intake on ovarian activity and fertility of heifers. Vet. Rec..

[60] Scharp D.W. (1979). Effect of adding superphosphate to the drinking water on the fertility of dairy cows. Aust. Vet. J..

[61] Cerosaletti P.E., Fox D.G., Chase L.E. (2004). Phosphorus reduction through precision feeding of dairy cattle. J. Dairy Sci..

[62] Tallam S.K., Ealy A.D., Bryan K.A., Wu Z. (2005). Ovarian activity and reproductive performance of dairy cows fed different amounts of phosphorus. J. Dairy Sci..

